# Preliminary report: parasympathetic tone links to functional brain networks during the anticipation and experience of visceral pain

**DOI:** 10.1038/s41598-018-31522-2

**Published:** 2018-09-07

**Authors:** James K. Ruffle, Steven J. Coen, Vincent Giampietro, Steven C. R. Williams, Qasim Aziz, Adam D. Farmer

**Affiliations:** 1 Centre for Neuroscience and Trauma, Blizard Institute, Wingate Institute of Neurogastroenterology, Barts and the London School of Medicine & Dentistry, Queen Mary University of London, 26 Ashfield Street, London, E1 2AJ UK; 2 Research Department of Clinical, Educational and Health Psychology, University College London, Gower Street, London, WC1E 6BT UK; 3 King’s College London, Institute of Psychiatry, Psychology & Neuroscience, Department of Neuroimaging, London, SE5 8AF UK; 4 Institute of Applied Clinical Sciences, University of Keele, Keele, Staffordshire ST5 5BG UK

## Abstract

The mechanisms that underpin the anti-nociceptive effect of the parasympathetic nervous system (PNS) on visceral pain remain incompletely understood. We sought to describe the effect of resting parasympathetic tone on functional brain networks during the anticipation and experience of oesophageal pain. 21 healthy participants had their resting cardiac vagal tone (CVT), a validated measure of the PNS, quantified, and underwent functional magnetic resonance imaging during the anticipation and experience of painful oesophageal distention. The relationship between resting CVT and functional brain networks was examined using 11 hypothesis-driven nodes and network-based statistics. A network comprising all nodes was apparent in individuals with high resting CVT, compared to those with low CVT, during oesophageal pain (family wise error rate (FWER)-corrected *p* < 0.048). Functional connections included the thalamus-amygdala, thalamus-hypothalamus, hypothalamus-nucleus accumbens, amygdala-pallidum, pallidum-nucleus accumbens and insula-pallidum. A smaller network was seen during pain anticipation, comprising the amygdala, pallidum and anterior insula (FWER-corrected *p* < 0.049). These findings suggest that PNS tone is associated with functional brain networks during the anticipation and experience of visceral pain. Given the role of these subcortical regions in the descending inhibitory modulation of pain, these networks may represent a potential neurobiological explanation for the anti-nociceptive effect of the PNS.

## Introduction

The autonomic nervous system (ANS) integrates the external environment with the internal milieu and serves to maintain bodily homeostasis, as well as the modulation of visceral nociceptive experience^1^. The ANS is conventionally considered to be composed of two branches, referred to as the parasympathetic (PNS) and the sympathetic nervous systems (SNS), and are considered to be broadly anti- and pro-nociceptive respectively^2–4^. Chronic visceral pain is common, occurring in both organic and functional gastrointestinal (GI) disorders, yet current treatment modalities are sub-optimal and not universally efficacious^5^. Thus, improvements in our understanding of the ANS’ influence on pain perception is needed, potentially leading to the therapeutic use of the anti-nociceptive effect of the PNS.

The vagus nerve is the primary neural substrate of the PNS and, with the SNS, forms a central component of the ‘brain-gut axis’^6^. Afferent vagal fibres arise from the mucosal and muscular layers of the GI tract and have their cell bodies in the nodose ganglia, which relay sensory information to the nucleus tractus solitarii (NTS) in the area postrema^7^. This is in close proximity to the dorsal motor nucleus (DMNx) and forms the ‘dorsal vagal complex’. From the dorsal vagal complex, visceral information ascends to subcortical areas, including the hypothalamus, thalamus and amygdala via the parabrachial nucleus which in turn relays information to higher cortical regions. These subcortical and cortical interconnections form the ‘central autonomic network’ which can modulate visceral function. In contrast, efferent vagal nerve fibres arise in the DMNx and nucleus ambiguus, innervating the foregut, midgut, and aspects of the hindgut^8,9^.

Given the central role of the vagus nerve within the brain-gut axis, it is not surprising that it has been implicated in the pathophysiology of a number of GI disorders characterised by chronic visceral pain, including inflammatory bowel disease and irritable bowel syndrome, where alterations in brain structure and function have previously been demonstrated^10–13^. We have recently reported that such alterations in the brain’s subcortical structure may be in-part attributable to the variance, or indeed disturbance, of autonomic function^14^, raising the prospect that these brain differences in visceral pain disorders may be attributable to differences in autonomic neurophysiology.

Although several studies have sought to exploit the therapeutic anti-nociceptive potential of PNS modulation, the neurophysiological and brain mechanisms of how one’s resting PNS functionality modulates their central processing of visceral pain in humans remains incompletely understood^2,15–20^. Pain is processed by many brain regions interacting as a network, so as to afford the stimulus not just sensory discrimination, but more complex cognitive, emotional and additionally pain-inhibitory attributes^21^. To date however, there is a paucity of visceral pain studies investigating brain function as a network. Thus, in the current study, we tested the specific hypothesis that *resting* PNS tone, quantified non-invasively using cardiac vagal tone (CVT), is associated with functional brain networks in the experience and anticipation of visceral pain.

## Methods

### Study Population

Thirty healthy participants (16 male and 14 female; mean age 30 years [range 21–53]; all right handed (assessed using the Edinburgh Handedness Inventory^22^) were recruited by local advertisement. Nine subjects (6 male and 3 female; mean age 29 years [range 23–39]) necessitated exclusion from analysis on the grounds of incomplete/artefactual neurophysiological or functional magnetic resonance imaging (*f*MRI) datasets: 3 due to artefactual disruption in electrocardiographic (ECG) traces which precluded accurate measurement of CVT, and 6 due to *f*MRI movement and/or noise artefacts which would otherwise confound brain networks analysis. Data was therefore subsampled to use the remaining twenty-one healthy participants (10 male and 11 female, mean age 30 years [range 21–53]).

Twenty-four hours prior to the study visit, all participants were asked to refrain from alcohol, caffeine and cigarettes, with female participants only studied during the follicular phase of their menstrual cycle in order to limit potential confounding factors of resting autonomic activity^23,24^. As the study investigated visceral pain, a previous history of GI symptoms; any visceral or somatic chronic pain disorders; neurological or endocrine disorders; any other chronic medical condition for which investigation or treatment was being sought and/or concurrent medication formed key exclusion criteria (in addition to MRI exclusion criteria). Aspects of the experimental data have been published previously by our group^25^, though for entirely disparate analytical investigations which are not discussed in this manuscript.

### Main Measures

#### Psychological factors

All participants first completed validated psychophysiological questionnaires, in order to limit for potential confounds on brain data and resting CVT^26^. The Hospital Anxiety and Depression Scale (HADS)^27^ was used to screen for subclinical anxiety and depression. Participants were excluded if they scored ≥8 on either scale. The Spielberger State-Trait Anxiety Inventory (STAI) was used to assess both *trait* and *state* anxiety^28^. State anxiety was evaluated prior to both autonomic data acquisition and MRI-scanning to prevent an anxiety confound that may not have been determined by the HADS-screening. The personality traits of extraversion, neuroticism and psychoticism were determined by the Eysenck Personality Questionnaire-Revised (EPQ-R)^29^.

#### Autonomic Measures

Autonomic parameters were recorded in accordance with international recommendations^30^. ECG electrodes (Ambu Blue Sensor P, Denmark) were placed at the cardiac apex, left and right sub-clavicular areas of each participant for ECG signal acquisition. ECG readings were digitally recorded using a bio-signals acquisition system (Neuroscope, Medifit Instruments, Enfield, Essex, UK) at 5 kHz. For each participant, mean digital arterial blood pressure (MBP) was measured non-invasively using a validated photoplethysmographic technique (Portapres, Amsterdam, Netherlands)^31^. Resting autonomic activity were derived by validated non-invasive cardiometrics; PNS activity by CVT, a putative measure of efferent brainstem vagal tone, and SNS by cardiac sympathetic index^1^. All individuals were studied in the afternoon (between 14:00–16:00) in a temperature controlled (20–22 °C), constantly lit, quiet laboratory. Participants were reclined at 45° on a bed with their legs supported. After attachment of all autonomic recording apparatus, a 20-minute recording period was undertaken. Participants were asked to relax, but were told not to fall asleep, during which resting autonomic tone was derived.

#### Parasympathetic Nervous System: Derivation of Resting Cardiac Vagal Tone

The derivation of resting parasympathetic activity by CVT is described in detail elsewhere^1^. In brief, the incoming QRS complex is compared to a unique template, generated from the initial ECG acquisition stages of the individual. If the QRS complexes are sufficiently comparable, voltage gated oscillators within the Neuroscope generate a 1 mV pulse, which feeds to a two-limb circuit, consisting of a high-pass and low-pass limb. The high-pass limb precisely follows the incoming QRS signal, whilst the low-pass limb produces a damped rendition^32^. Therefore, the lesser the delta change of an incoming signal, that is to say the lower the heart rate variability (HRV), the closer the low-pass limb will mimic that to the high-pass, resulting in a lower value. In contrast, the greater the HRV the more the low-pass limb will deviate from its high-pass counterpart, resulting in a higher value. This phenomenon is referred to as ‘phase shift demodulation’ and is uniquely based upon non-invasive measures of PNS tone. CVT is measured on a linear vagal scale (LVS), where a value of 0 is derived from fully atropinized healthy human volunteers^1,33^. Moreover, mathematically CVT correlates closely to other putative ‘parasympathetic’ measures, including both HRV and root mean square of successive differences (RMSSD)^34^.

#### Sympathetic Nervous System: Cardiac Sympathetic Index

Resting sympathetic nervous system (SNS) activity was quantified by means of the cardiac sympathetic index (CSI), so as to ensure there was no further autonomic confound when investigating the relationship between vagal tone and brain data^14^. To determine CSI, R-R interval data was first extracted from the ECG and manually reviewed to remove any artefacts. Subsequently, R-R data was transferred to the cardiac metric program, which yields a calculation of the validated Toichi’s cardiac sympathetic index^35^. Notably, CSI is a numerical ratio of R-R intervals and thus has no units^36^.

### Main Exposures

#### Painful Visceral Stimulation

A visceral pain stimulus was employed by mechanical oesophageal distension. The original development and validation of this stimulus is described elsewhere^37^. Prior to MRI scanning, a 3-mm nasogastric catheter (Sandhill Scientific, Oxford, UK), with a 2-cm air-distensible silicone balloon mounted to the distal tip, was positioned trans-nasally in the distal oesophagus without the aid of local anaesthetic, such that the midpoint of the balloon was 35 cm *ab nares*. The nasogastric catheter was connected to an MRI-compatible, purpose-built pump (Medical Physics Department, Hope Hospital, Salford, England), capable of rapid balloon distension (maximum flow rate, 200 millilitres per second (mL/s); rise time to maximum balloon inflation, 165 milliseconds for any given pressure). Inflation pressure ranged from 0 to 35 psi, with stimulus frequency of 0.3 Hz. The air pressure needed to inflate balloon to the pain tolerance threshold (PTT) was defined individually in each participant, whereby the volume of distension was increased in 2 mL increments (from 0) until volunteers stated they could no longer tolerate a further increase in the pain stimulus, hence their PTT. This approach allows for individual differences in oesophageal anatomy between participants.

#### *f*MRI Experimental Paradigm

An event-related design with three distinct epochs was used: (i) visceral pain anticipation, (ii) mechanical oesophageal pain and (iii) safety from pain, allowing time for participants to return to near baseline. The *f*MRI experiment consisted of 20 trials incorporating each of the 3 epochs as above, thus exposing each participant to a total of 60 events. To instigate the anticipation of pain, but also safety from pain, a visual cue program was used, the details of which were described to participants prior to MRI scanning. Each trial began with the presentation of a visual warning cue by means of a coloured square, which would signify a painful visceral stimulus was imminent, hence inducing pain anticipation. Following this, the painful stimulus would be delivered for 1 second whilst scanning continued. Immediately afterwards, a second differently coloured square would be displayed to signal safety from pain.

**Figure 1:**
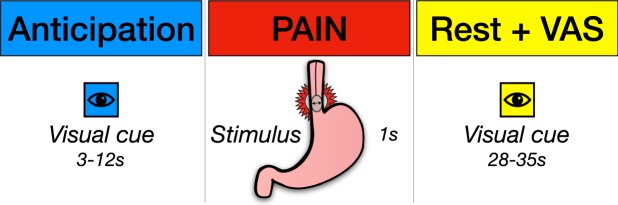
Time-course of a single experimental trial The *f*MRI experiment was conducted in an event-related design of 3 epochs. These were (i) anticipation of pain, (ii) visceral pain by means of mechanical oesophageal stimulation, and (iii) safety (from stimulation), where a subject would also give a pain rating (by use of VAS). This was repeated 20 times per subject, whereby the durations of the anticipation and safety events varied throughout to prevent any conditioning which could influence experimental findings. Cue colours were randomised to prevent any colour/condition pairing confound. Abbreviations: S, seconds; VAS, visual analogue scale.

The timing of each condition of the event-related design was pseudo-randomised and jittered to the TR (repetition time) in order to avoid habituation and enable a representative sample of the brain response during each condition. The anticipation period signalled the start of a new trial and lasted *between* 3–12 seconds, whilst the ‘safe’ phase lasted between 28–35 seconds, commencing 9–15 seconds after the onset of painful stimulation. During the safety period, participants was asked to rate the perceived intensity of the pain they experienced using a VAS scale and had 5 seconds to do so. This VAS-rating was on a 100-point scale, whereby 0, 50 and 100 represented no sensation, mild discomfort and extreme pain respectively. A further stimulus free period of between 8–21 seconds followed this prior to the commencement of the next trial. The use of coloured visual cues for the anticipatory and safety events was counterbalanced to avoid any possible confounding effects of colour plus experimental condition pairing; such that half of the participants received a blue square for the anticipatory cue with yellow for safety, whilst the other half received a yellow square for the anticipatory cue and blue for safety. The variable-onset timing of both pain anticipation and pain *proper* was pseudo-randomized to increase the unpredictability of stimuli so as to reduce the effects of event habituation, thus increasing the efficacy of the anticipatory period (see Fig. 1).

#### MRI Data Acquisition

MRI data were acquired using a General Electric (Milwaukee, WI, USA) SIGNA HDx 3.0 Tesla MR scanner, fitted with an 8-channel, phased-array receive-only head coil, located at the Centre for Neuroimaging Sciences, Institute of Psychiatry, Psychology & Neuroscience, King’s College London. Head movement was minimised by application of foam padding within the head coil. During scanning, participants could view a screen that would project the aforementioned visual cues signifying the pain stimulus or the safety period. Participants recorded their individual VAS pain ratings by means of an MRI-compatible button box that would be placed in the right hand of each participant.

#### Scanning Parameters

For each subject, a high resolution T1-weighted 3D FSPGR structural image was acquired for normalisation of the fMRI data to standard space in sagittal orientation using the following parameters: repetition time (TR) = 7.02 ms; echo time (TE) = 2.82 ms, inversion time (TI) = 450 ms; slice thickness = 1.1 mm; field of view = 280 mm; flip angle = 20°; spatial positions = 196; image matrix = 256 × 256 × 196 voxels; in-plane voxel dimensions = 1.1 × 1.1 × 1.1 mm. For functional brain data, multi-slice T2*-weighted *f*MRI BOLD contrast images were acquired sequential to the structural scan, with the following parameters: 40 × 3 mm slices (imaging whole brain from cerebellum to vertex); TR = 2500 ms; TE = 30 ms; slice thickness = 3 mm; interslice gap = 0.3; flip angle = 80°; in-plane resolution = 64 × 64; in-plane voxel dimensions = 3.75 × 3.75 mm^2^; number of volumes = 480.

#### Pre-Processing of *f*MRI Data

*f*MRI data pre-processing was undertaken using FMRI Expert Analysis Tool (FEAT) version 5.98, part of the FMRIB Software Library (FSL) software package (www.fmrib.ox.ac.uk/fsl)^38^. The following pre-statistics processing was applied; Motion Correction Linear Image Registration Tool (MCFLIRT) (7 degrees of freedom); slice-timing correction using Fourier-space time-series phase-shifting; brain extraction (BET); spatial smoothing using a Gaussian kernel of full-width-half-maximum (FWHM) 5 mm; grand-mean intensity normalisation of the entire 4-dimensional dataset by a single multiplicative factor; high pass temporal filtering (Gaussian-weighted least-squares straight line fitting, with sigma = 50.0 s). Gaussian smoothing of 5 mm was chosen given (i) relatively small sizes of brain region of interests (ROIs) and (ii) practice adopted and advocated by other groups^38,39^. Registration to high resolution structural and standard space images was carried out using the FMRIB Linear Image Registration Tool (FLIRT).

#### Brain Regions of Interest

Brain regions previously implicated in *both* visceral pain and the regulation of parasympathetic tone were selected *a priori* from current literature, including that of previous findings from within our group on different subjects (see refs^7,14,40–45^). Based upon this, 11 regions of interest (ROIs) were selected, comprising the following: (i–ii) left and right amygdala; (iii–iv) left and right anterior insula cortex; (v–vi) left and right NAc; (vii–viii) left and right pallidum; (ix–x) left and right thalamus; and (xi) hypothalamus.

The eleven structural masks for each anatomical ROI were generated using the 2 mm Montreal Neurological Institute (MNI) 152 T1 template brain within FSL-view. For ROIs (i-x) the Harvard subcortical atlas was utilised, with probability thresholding set to >50% to prevent probability overlap, whilst for ROI (xi) the Talairach brain map was used as presently no Harvard map exists for this region. Moreover, this approach is in keeping with previous *f*MRI studies investigating the hypothalamus^46^.

BOLD signal was mapped to the experimental epochs, allowing the association of brain ‘activity’ during both pain anticipation and pain. BOLD signal during both pain and pain anticipation epochs were extracted from each region. For the pain epoch, a 5-second period were extracted to maximise extractable haemodynamic response following an acute pain stimulus. Using MATLAB (version 2016a, uk.mathworks.com), BOLD signal from each ROI, or ‘*node’*, was cross-correlated, producing correlation matrices of 55 node-node correlation (*r)* values, or ‘*edges’* (resultant of binomial coefficient $(\frac{11}{2})=55;\,{\rm{or}}\,11\,\mbox{''}choose\mbox{''}\,2)$.

#### Network Based Statistics

The Network Based Statistics (NBS) connectome toolbox (version 1.2 https://www.nitrc.org/projects/nbs/) was used to investigate brain network differences during pain and pain anticipation, contingent on an individual’s resting CVT^47^. The NBS is a non-parametric statistical method which corrects for multiple comparisons, and controls for the family-wise error rate (FWER). The NBS is the graph analogue of cluster-based statistical methods used in mass univariate testing on all pixels in an image. As opposed to clustering in physical space, the NBS clusters in topological space, whereby the most basic equivalent of a cluster is a graph component. The method permits derivation of FWER-corrected *p*-values using permutation testing when investigating brain networks with *f*MRI^48,49^.

The effect of resting CVT on brain networks during both anticipation and painful visceral stimulus was analysed by dichotomizing patients based upon a median split of resting CVT value, a paradigm previously used in a number of studies undertaking similar investigations, including with *f*MRI^50,51^. An additional rationale for this choice of median split is that previous work has demonstrated that pain endophenotype stratification demonstrates clustering into two disparate psychophysiological profiles, the strongest predictive factor of which was high or low parasympathetic tone (quantified by CVT)^3,25^. Thus, stratification of individuals to high or low resting parasympathetic CVT for pain may hold some academic and clinical benefit, for example for allocating an autonomic neuromodulation therapy. NBS analyses performed were *t-*tests, using 10,000 permutations, and the criteria for significance set to FWER-corrected *p* < 0.05. An edge parameter threshold of 1.658 was used as per previous similar studies^52^. For significant networks identified, the test was repeated with 50,000 permutations to clarify data remained significant and to maintain statistical stringency, following which *t* values were extracted and results visualized using the BrainNet illustrative package (http://www.nitrc.org/projects/bnv/)^53^.

### Statistics

Autonomic, demographic and psychophysiological data normality distributions were first visually inspecting histograms and were tested using the D’Agostino-Pearson omnibus K2 normality testing. All data were normally distributed, and hence throughout the article as mean ± SD. CVT-dichotomised groups were investigated with two-tailed *t*-tests to ensure no demographical, autonomic or psychological possible confounds on brain networks data existed. *P* < 0.05 was adopted as the criterion to indicate statistical significance. Statistical analyses were performed using MATLAB (version 2016a, uk.mathworks.com) and GraphPad Prism (version 6.00, GraphPad Software, La Jolla California, USA, www.graphpad.com). Statistical analysis of neuroimaging data was undertaken by permutation-tested NBS with FWER multiple comparison correction (see above).

### Study Approval

The study was approved by the Queen Mary, University of London ethics committee (ref CREC/07/08-7). Written informed consent was obtained from each participant after the nature and the purpose of the study had been explained. All subjects were naïve to the experimental protocol, and were remunerated for participation. All methods were performed in accordance with the relevant guidelines and regulations.

## Results

### Clinical Data

#### Baseline data

**Figure 2:**
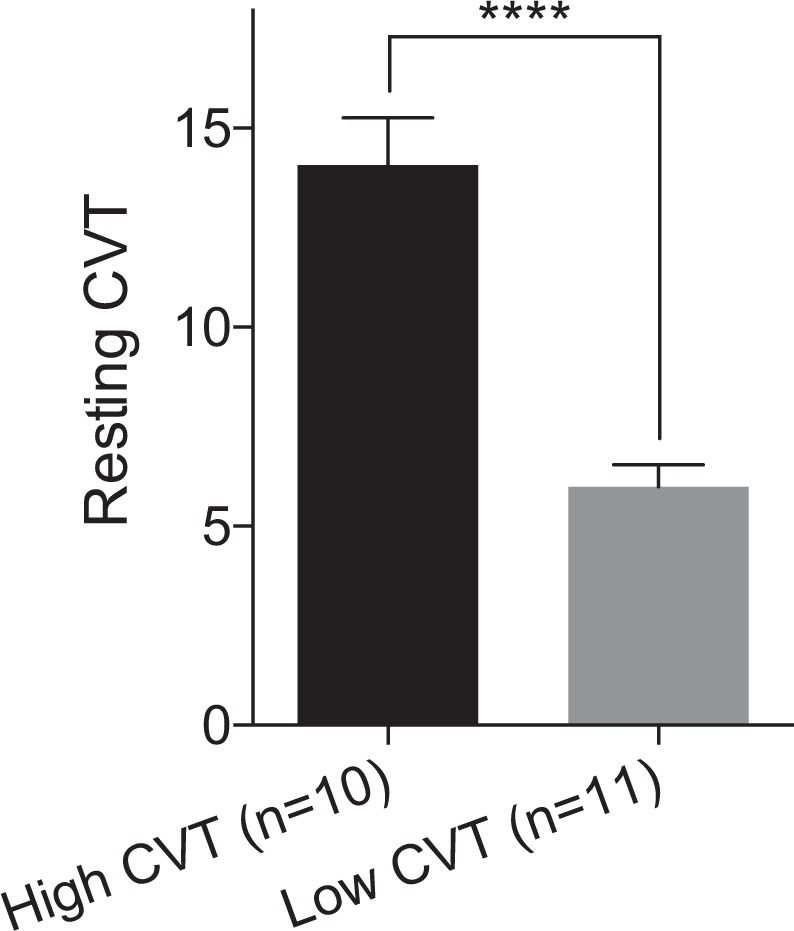
Group resting parasympathetic cardiac vagal tone For *f*MRI analysis, all subjects were dichotomised by means of a median split based upon the numerical magnitude of their resting CVT. The ‘high CVT’ group consisted of 10 subjects and the ‘low CVT’ group consisted of the remaining 11 individuals. As intended, the resting CVT of these groups significantly differed (****p < 0.0001), for later MRI analysis. Abbreviations: CVT, cardiac vagal tone.

Group cohort demographic, autonomic and psychophysiological data is shown in Table 1. In particular, resting CVT was 9.84 ± 5.02 and mean heart rate (HR) was 69 ± 11.58, which negatively correlated to resting CVT (*r* = −0.69, *p* < 0.0005). Mean resting CVT in the ‘high resting CVT’ group was 14.07 ± 3.76, whilst in the ‘low resting CVT’ group was 5.99 ± 1.84, which, as intended, significantly differed (*p* < 0.0001), *see* Fig. 2. All participants had a CVT recording within the published normal range^1^. Baseline mean systolic and diastolic blood pressure was 135 ± 38.33 and 66 ± 17.54, respectively. Baseline mean cardiac sympathetic index (CSI) was 2.18 ± 0.73. *During oesophageal pain*, mean visual analogue score (VAS) was 63.67 ± 11.77, with a mean PTT of 21.24 ml ± 6.66.

**Table 1:** Cohort demographical, psychological, autonomic and experimental pain/sensory data for all subjects (n = 21).

Demographic, Autonomic and Psychophysiological Differences				
**Variable**	**Full Cohort (n = 21)**	**Low resting CVT Group (n = 11)**	**High resting CVT Group (n = 10)**	**Difference Between Sub-groups**
***Demographical***				
Age (years)	29.86 ± 9.15	33 ± 11.59	27 ± 3.60	NSD (p = 0.11)
Gender (M/F)	10/11	6/5	4/6	NSD (p = 0.67)
***Personality Traits***				
Extraversion (0–23)	17.40 ± 3.66	17.40 ± 3.66	17.40 ± 3.86	NSD (p > 0.99)
Psychoticism (0–32)	5.65 ± 4.51	5.80 ± 4.73	5.50 ± 4.53	NSD (p = 0.89)
Neuroticism (0–24)	9.19 ± 6.46	10.45 ± 7.05	7.80 ± 5.77	NSD (p = 0.36)
***Hospital Anxiety & Depression Scale***				
Anxiety (0–10)	3.14 ± 2.39	3.27 ± 2.57	3.00 ± 2.31	NSD (p = 0.80)
Depression (0–10)	1.95 ± 1.80	2.18 ± 2.27	1.70 ± 1.16	NSD (p = 0.55)
***State Trait Anxiety Inventory***				
State (pre-Neuroscope) (20–80)	29.19 ± 7.44	28.64 ± 9.03	29.80 ± 5.63	NSD (p = 0.73)
State (pre-MRI) (20–80)	29.38 ± 6.30	28.36 ± 6.18	30.50 ± 6.55	NSD (p = 0.45)
Trait (20–80)	34.71 ± 10.12	36.91 ± 12.18	32.30 ± 7.09	NSD (p = 0.31)
***Autonomic Measurements***				
Resting HR (bpm)	68.80 ± 11.58	74 ± 11.05	63 ± 9.97	*p = 0.036
**Resting CVT (LVS)**	**9.84 ± 5.02**	**5.99 ± 1.84**	**14.07 ± 3.76**	****p ** < 0.0001**
Resting CSI (ratio)	2.18 ± 0.73	2.25 ± 0.79	2.10 ± 0.70	NSD (p = 0.64)
Systolic BP (mmHg)	135.40 ± 38.33	146 ± 31.6	118.2 ± 43.89	NSD (p = 0.16)
Diastolic BP (mmHg)	66.24 ± 17.54	70.46 ± 14.35	59.90 ± 21.26	NSD (p = 0.27)
***Oesophageal Sensory & Pain Quantifiers***				
Sensory Threshold (ml)	6.71 ± 5.69	6.27 ± 5.08	7.20 ± 6.55	NSD (p = 0.72)
Pain Tolerance Threshold (ml)	21.24 ± 6.66	22.64 ± 7.50	19.70 ± 5.58	NSD (p = 0.33)
VAS (0–100)	63.67 ± 11.77	61.45 ± 10.23	66.10 ± 13.39	NSD (p = 0.38)

Variables herein are described as mean ± standard deviation. CVT lies on a linear vagal scale and thus has no units, whilst resting CSI is expressed as a ratio of R-R intervals. Only resting parasympathetic cardiac vagal tone (CVT) and resting heart rate (HR) significantly different between groups, to which CVT is derived from computation of HR tracing and thus a difference would be expected. Abbreviations: BP, blood pressure; BPM, beats per minute; CVT, cardiac vagal tone; F, female; M, male; SD, standard deviation; LVS, linear vagal scale; VAS, visual analogue scale.

All demographic, psychophysiological cardiovascular, pain-metric and autonomic [other than CVT] data were compared between the resting CVT groups to ensure no group confounders existed. None of these measures were significantly different between the two groups, apart from resting HR as expected, see Table 1. Neither PTT nor VAS significantly differed between groups, suggestive of a relatively comparable pain stimulus between both groups, although actual balloon inflation differed between participants.

### Functional Connectivity

**Figure 3:**
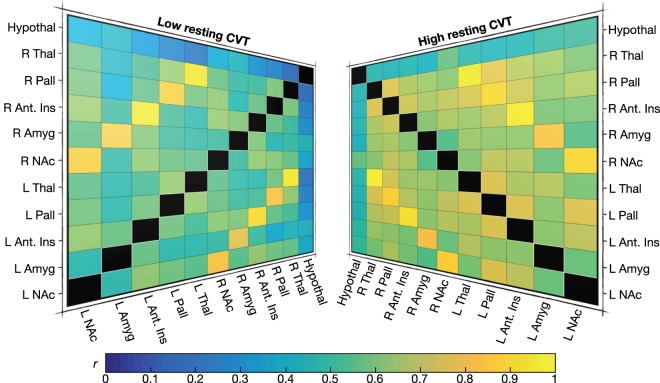
Group-wise oesophageal pain connectivity Firstly, ROI-driven connectivity matrices were generated for each of the 21 subjects, by a *prior* selection of 11 nodes. For illustrative purposes, here we show mean correlation (*r*) ‘colour-maps’ of connectivity in both the low (left) and high (right) resting CVT groups during acute oesophageal pain. A colour bar at the bottom of the figure describes the colour by which a particular correlation value is assigned, whereby a bluer square corresponds to a poorly connected pair of nodes (for example – for the low resting CVT group, the hypothalamus and right thalamus functional connection), whilst a more orange or yellow square corresponds to a highly connected pair of nodes (for example, for the high resting CVT group, the right and left thalamus functional connection). Values assigned to colour are that of correlation or *r* values. Abbreviations: Amyg, amygdala; Ant, anterior; CVT, cardiac vagal tone; Hypothal, hypothalamus; Ins, insula cortex; L, left; NAc, nucleus accumbens; Pall, pallidum; R, right; ROI, region of interest; Thal, thalamus.

Correlation matrices were generated from 11 nodes by blood-oxygen-level dependent (BOLD) contrast (~equivalent to brain region *activity*), equating to a connectivity network of 55 edges for both pain and pain anticipation. Neither brain activity nor pairwise connectivity was compared statistically between the high and low CVT groups, given our prior hypothesis specific to network-based connectivity^47^. For illustrative purposes however, we provide a visual representation of pairwise connectivity differences for all 55 edges between both the low and high resting CVT groups during pain (*see* Fig. 3). Notably, pairwise connectivity between left and right equivalent brain regions is high during visceral pain, for instance activity in the left and right amygdala is notably highly correlated, as is activity between the left and right thalamus.

### High Resting Cardiac Vagal Tone is Associated with a Functional Brain Network During the Experience of Visceral Pain

**Figure 4:**
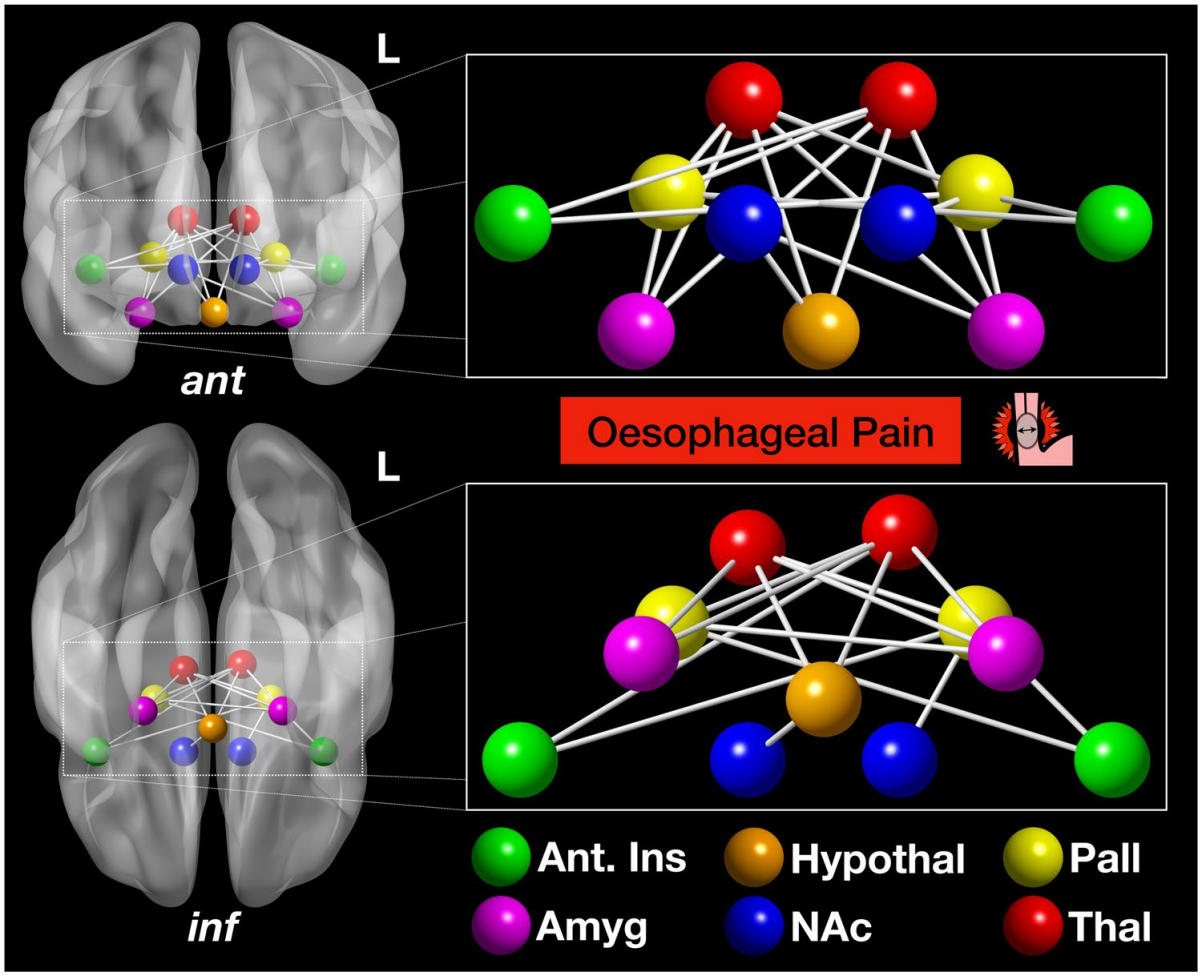
High resting vagal tone corresponds to a subcortical functional network during oesophageal pain A significant subcortical network comprising 11 nodes and 18 edges was apparent in the high resting CVT group, compared to the low resting CVT group, during acute oesophageal pain (FWER-corrected *p* < 0.048). These nodes (coloured spheres) and edges (grey lines) are illustrated above. The top image shows the anterior-coronal view of this network, whilst the bottom image illustrates the inferior-axial view. The left side of brain is demarcated by L. Nodes are color-coded as per the colour key. Abbreviations: Amyg, amygdala; Ant, anterior; Hypothal, hypothalamus; Inf, inferior; Ins, insular cortex; L, left; NAc, nucleus accumbens; NBS, network based statistics; Pall, pallidum; Thal, thalamus.

**Figure 5:**
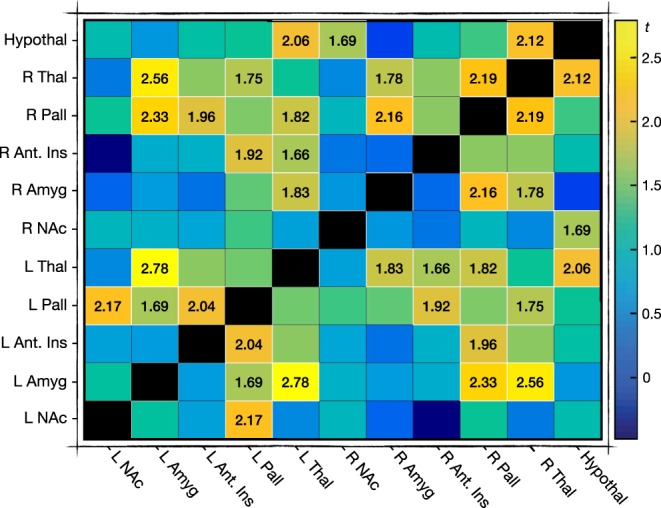
Significant network edges of the oesophageal pain-high resting vagal tone network A significant subcortical network comprising 11 nodes and 18 edges in the high resting CVT group, during acute oesophageal pain, was identified (FWER-corrected *p* < 0.048). This network is anatomically demonstrated in Fig. 4, but here we illustrate the edge weights (~the magnitude of edge strength by means of the NBS primary thresholding, or *t*). The colour-map shows the *t* strengths for each connection between two ROI nodes. Edges of the high resting CVT group that were statistically significantly greater than of the low resting CVT group are highlighted on the colour-map by recording of the actual *t* value. The colour of squares is determined by means of the numerical value of *t*, illustrated by the colour-bar on the right side of the image. Abbreviations: Amyg, amygdala; Ant, anterior; CVT, cardiac vagal tone; Hypothal, hypothalamus; Ins, insula cortex; L, left; NAc, nucleus accumbens; Pall, pallidum; R, right; ROI, region of interest; Thal, thalamus.

**Figure 6:**
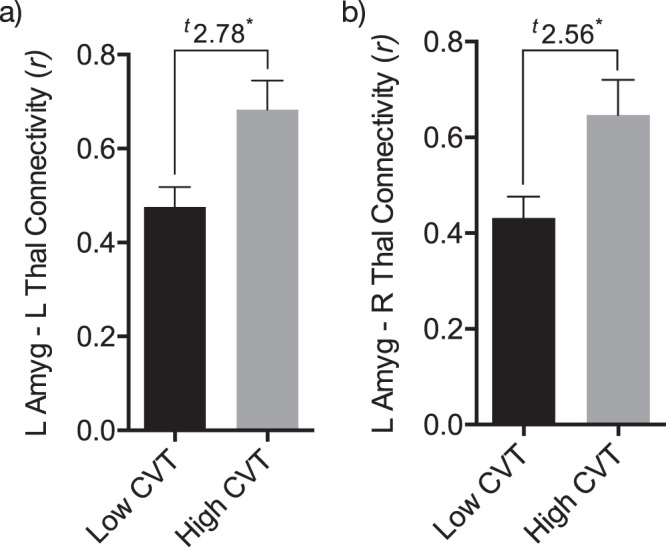
Thalamus-amygdala connectivity during visceral pain significantly differs contingent on resting parasympathetic tone Connectivity between the bilateral thalamus and left amygdala were the edges most significantly different between the low and high CVT subgroups. (**a**) L amygdala – L thalamus CVT group connectivity; (**b**) L amygdala – R thalamus connectivity. T-stat values for these edges, quantified by the NBS, are depicted atop. Significance is determined for the network as a whole to accommodate for FWER multiple comparison correction. Abbreviations: CVT, cardiac vagal tone; FWER, family-wise error rate; L, left; NBS, network based statistics, R, right.

A significant functional brain network was apparent in the high resting CVT group, compared to the low resting CVT group (i.e. *t*-test of high resting CVT group > low resting CVT group). The network comprised 11 brain nodes, with 18 edges (~functional network connections) (FWER-corrected *p* < 0.048). These specific functional connections were numerous and thus are best viewed in visual representation, *see* Fig. 4 and Movie 1. Specifically, significant edges included the following: left (L) nucleus accumbens (NAc) – L pallidum, bilateral amygdala – bilateral thalamus, L amygdala – bilateral pallidum, L anterior insula – bilateral pallidum, L pallidum – right (R) thalamus, L pallidum – R anterior insula, hypothalamus – bilateral thalamus, hypothalamus – R NAc and R anterior insula – L thalamus. Edge weights of specific functional connectivity differences between the high and low resting CVT groups can be visualised in the colour map, *see* Fig. 5. Notably, the two most significantly differing edges were that of L amygdala – L thalamus and L amygdala – R thalamus, *see* Fig. 6. In contrast, no significant network was identified for the low resting CVT group, in comparison to the high resting CVT group (i.e. *t*-test of low resting CVT group > high resting CVT group).

### High Resting Cardiac Vagal Tone Individuals Exhibit a Subcortical Functional Network During the Anticipation of Visceral Pain

**Figure 7:**
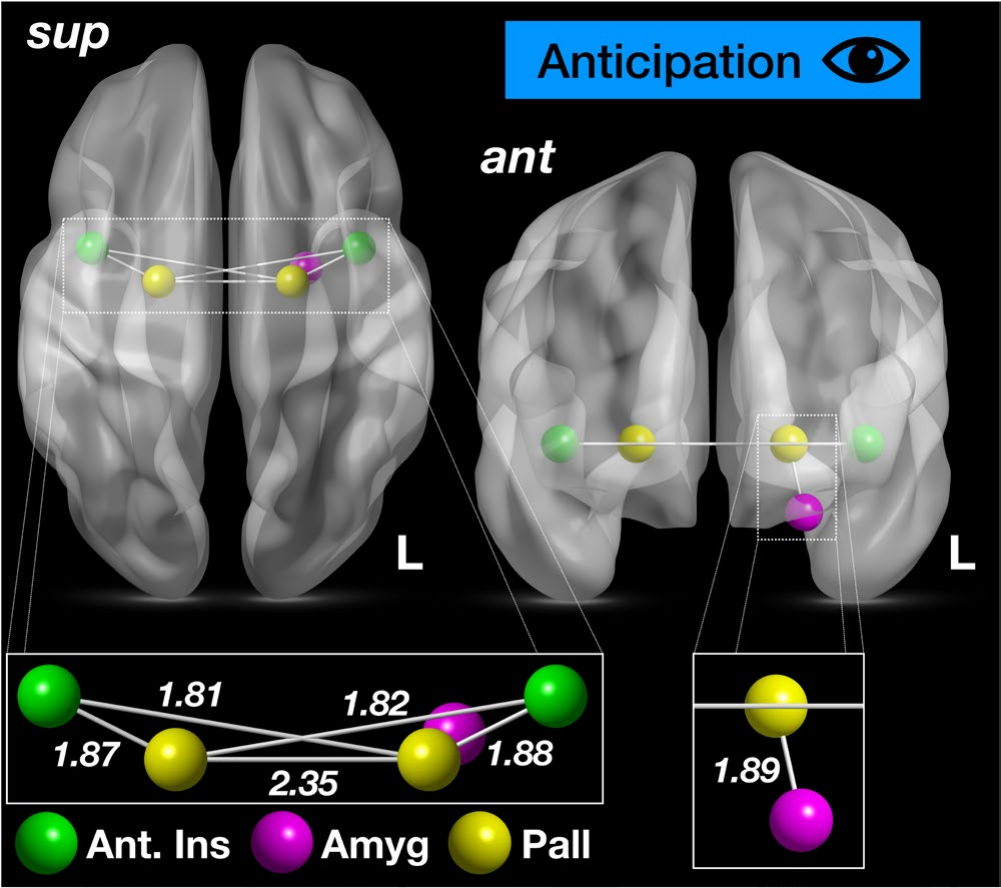
High resting vagal tone corresponds to a subcortical functional network during anticipation of pain A significant subcortical network comprising 5 nodes and 6 edges in the high resting CVT group was apparent during anticipation of pain (FWER-corrected *p* < 0.049). These nodes (coloured spheres) and edges (grey lines) are illustrated above. The left image shows the superior-axial view of the network, whilst the right image shows the anterior-coronal view. The left side of brain is demarcated by L. Nodes are color-coded as per the colour key. Furthermore, edge weights (*t* values of the NBS network) are recorded in the lower aspects of the images (without superimposed structural brain scan). Abbreviations: Amyg, amygdala; Ant, anterior; L, left; Pall, pallidum; Sup, superior; Thal, thalamus.

We subsequently investigated whether high resting CVT and a visceral-pain specific network (or indeed just part of it) was apparent during the anticipation of visceral pain. Interestingly, this revealed a significant (yet condensed) subcortical network, again demonstrable in the high resting CVT group when compared to the low resting CVT group (i.e. *t*-test of high resting CVT group > low resting CVT group). This functional network comprised 5 nodes and 6 edges (FWER-corrected *p* < 0.049), *see* Fig. 7 and Movie 2. The significant edges were: bilateral anterior insula – bilateral pallidum; L pallidum – L amygdala and L pallidum – R pallidum. The edge weight differences between both the high and low resting CVT groups for these functional connections is shown in Fig. 7. No significant network was apparent for the low resting CVT group in comparison to high resting CVT during pain anticipation (i.e. *t*-test of low resting CVT group > high resting CVT group).

## Discussion

We provide novel preliminary evidence which suggests that differences in resting PNS tone is associated with functional brain networks involved in processing the anticipatory and experiential components of visceral pain. Specifically, we demonstrate that higher resting PNS tone is associated with a subcortical network, which develops in part during the anticipation of visceral pain (the threat-arousal response), but becomes fully established during visceral pain. During the anticipation phase, this network comprised the anterior insula cortex, amygdala and pallidum (Fig. 7, Movie 2), but during pain it extended to include the thalamus, NAc and hypothalamus (Figs 4–6, Movie 1). These findings have a number of implications.

### High Resting Cardiac Vagal Tone and the Effect on Pain-Based Brain Networks

Although it has been inferred that the brain regions investigated in our study are involved both in visceral pain processing *and* parasympathetic regulation (see refs^7,14,40–44^), our study is the first to provide objective data to confirm this. Similarly, neither the concurrent interaction between these roles nor the communication between these regions has been previously objectively studied using a networks-based analytical approach.

A diverse array of factors such as gender, ANS, personality traits and genotype influence the brain processing of visceral pain and these factors interact to influence pain sensitivity^3,25,51,54,55^. For instance, Farmer *et. al* demonstrated that resting parasympathetic CVT and personality form critical factors attributable to the clustering of individuals as specific pain ‘endophenotypes’^3,25^. Those with high resting CVT, extrovert personality and the L allele of the serotonin transporter genotype were less pain sensitive compared to those with low resting CVT, neurotic personality and S allele of the serotonin transporter genotype. Our current data demonstrate that a network of functional connectivity, which represents the extent of information transmission between these brain regions^48,49^, is significantly greater in high resting CVT individuals compared to low resting CVT individuals during both the threat of visceral pain and its actual experience. This high resting CVT associated network is composed of numerous edges (singular functional connections between two brain regions) which require further discussion.

The thalamus is a key region of the homeostatic-afferent pain network and is important for visceral pain processing^56^. Moreover, it forms a central node of the high resting CVT-based network found, with multiple bilateral spanning edges to regions such as the anterior insula, amygdala and hypothalamus. In these individuals, the thalamus displayed greater functional connectivity to the *right anterior* insula, an area especially associated with visceral pain *and* interoception^57^. These results are in keeping with the previously characterised parasympathetic-derived homeostatic afferent pathway, where PNS afferents synapse at the NTS before relaying to thalamic nuclei and in turn to the anterior insula, a region which some have referred to as the ‘foundation for subjective human feelings’^58,59^.

The identified network also illustrated greater functional connectivity between the thalamus and amygdala, and between the thalamus and hypothalamus. The amygdala is an important component of the central autonomic network and forms part of the emotional-arousal network in the context of pain^7^. Moreover, the two most significant edges of the network connected the left and right thalamic nodes to the left amygdala. The multiple functions of the hypothalamus include the coordination of autonomic homeostasis^7^. We posit our findings of increased amygdala-thalamic-hypothalamic connectivity may be a functional representation of the recently characterised structural white matter amygdalofugal pathway, suggested to relay information from the amygdala to the thalamus and onwards to the hypothalamus^60^. We postulate that these strengthened functional connections in high resting CVT individuals during oesophageal pain may represent firstly a greater pain-emotional arousal response, but secondly a greater transfer of visceral pain-encoded information from the amygdala to the thalamus and hypothalamus (via the amygdalofugal pathway), which in turn would influence the hypothalamic control of autonomic homeostasis^7,60^.

The pallidum was also part of the network reported in high resting CVT individuals. This basal ganglia nucleus has documented roles in both somatic pain processing and autonomic regulation^14,43^, however, it is an understudied region in the context of visceral pain. The basal ganglia pain-specific connectivity patterns are numerous and, overall, the pallidum is thought to be involved in the integration of information between brainstem, subcortical and cortical regions, influencing the sensory, emotional/cognitive and endogenous/modulatory domains of pain processing^43^. We thus propose an increased engagement of pathways related to these pain domains in participants with higher resting CVT.

### High Resting Cardiac Vagal Tone and Brain Networks of Visceral Pain Anticipation

During pain anticipation, a condensed version of the oesophageal pain network in high resting CVT participants was apparent, with functional connections including the insula-pallidum, pallidum-pallidum and pallidum-amygdala. The roles of these specific nuclei and pathways in pain have been discussed in detail above. However, their functional interaction and information transfer during the threat of visceral pain may be indicative of the emotional arousal response to impeding pain or threat, including a sense of a disrupted self-wellbeing/interoception.

### Limitations

This study is not without limitation. Firstly, the cohort size is small, thus the power for statistical analysis is reduced. However, many known confounding factors are controlled for in this study and the sample size is comparable to other studies utilising similar analysis (see^23–26,51,52,54,55^), such that after permutation testing and FWER-correction significant results have still been yielded for this preliminary report. Secondly, the oesophageal stimulus we utilised was not designed to evaluate differences in pain severity contingent on a given variable (e.g. CVT), but rather to cause an equivalent amount of pain/distress to all participants. This was to ensure that brain analysis was not confounded by differences in stimulus-severity relative to factors, such as oesophageal diameter. Future studies should categorically interrogate this issue. Pertaining to the analysis method, we opted for a hypothesis-driven ROI approach, however it should be noted that numerous alternative analysis methods are possible, such as with independent component analysis, dual regression or otherwise, which further studies should investigate. In addition, measurement of resting CVT was undertaken in our controlled laboratory environment on a different day to MRI scanning. The rationale for this was to ascertain as close to ground truth as possible a recording of ‘baseline’ CVT. This was not taken prior to MRI, or indeed during MRI, as it is likely that MRI noise, stressful environment or otherwise could have otherwise confounded a ‘resting’ PNS quantification. Lastly, our experimental aim in this particular study was to investigate how *resting* CVT might predispose an individual to process visceral pain disparately, in keeping with aforementioned pain endophenotyping studies^3,25^. Future studies to investigate *real-time* changes to CVT during pain with concomitant neuroimaging would additionally be beneficial to further one’s understanding of autonomic neurophysiology and pain processing (see^18^).

### Implications for Future Studies and Conclusions

Our study provides novel evidence of the interplay between parasympathetic CVT and the brain processing of both visceral pain and the threat arousal that precedes it. Given that clinical disorders such as irritable bowel syndrome are encompassed by visceral pain and autonomic imbalance^10,61^, the data may reveal the underpinning neural mechanism to this. The study highlights that parasympathetic tone is an important factor in the inter-individual variability of pain processing in the brain, which should therefore be controlled for in neuroimaging studies. Given that numerous previous studies have identified that differences in parasympathetic tone accompany changes in pain perception, both in health and disease, it is possible that this network reflects an autonomic influence on the endogenous pain modulation pathway. This raises the prospect of treating chronic visceral pain disorders in a clinical setting using novel non–pharmacological methods for increasing parasympathetic tone, the proof of concept for which was recently provided by our study demonstrating that slow deep breathing attenuates acid induced oesophageal hypersensitivity in healthy subjects^2^. Based on our findings, we question if parasympathetic modulation may thus influence a central brain network of pain processing. Further study is warranted to concomitantly neuroimage the effect of autonomic neuromodulation by an experimental increase in PNS tone, such as by electrical transcutaneous vagal nerve stimulation, which has shown efficacy in visceral pain^15–18^. Further studies are also warranted to investigate whether network-based connectivity can be used as a biomarker for pain sensitivity, visceral hypersensitivity and perhaps even for therapeutic efficacy in autonomic neuromodulation.

## Electronic supplementary material

Supplementary Information

Movie 1: High resting vagal tone corresponds to a subcortical functional network during oesophageal pain

Movie 2: High resting vagal tone corresponds to a subcortical functional network during anticipation of pain

## Data Availability

Autonomic data used in this study is available upon reasonable request.
