# Supplementary material for: Preliminary report: parasympathetic tone links to functional brain networks during the anticipation and experience of visceral pain
Source: Sci Rep. 2018 Sep 7;8:13410. doi: 10.1038/s41598-018-31522-2 (PMC6128833; doi:10.1038/s41598-018-31522-2)
Supplement: Supplementary file 1 — Supplementary Information [file 41598_2018_31522_MOESM1_ESM.docx]

**SUPPLEMENTARY INFORMATION FILE**

**3D MOVIE LEGENDS [For Online Version]**

**Movie 1: High resting vagal tone corresponds to a subcortical functional network during oesophageal pain – 3D Movie**

A significant subcortical network comprising 11 nodes and 18 edges was apparent in the high resting CVT group, compared to the low resting CVT group, during acute oesophageal pain (FWER-corrected *p*<0.048). These nodes (coloured spheres) and edges (grey lines) are illustrated here in a 3-dimensional rotational movie. Nodes are color-coded as per the colour key. Abbreviations: Amyg, amygdala; Ant, anterior; Hypothal, hypothalamus; Inf, inferior; Ins, insular cortex; L, left; NAc, nucleus accumbens; NBS, network based statistics; Pall, pallidum; Thal, thalamus.

**Movie 2: High resting vagal tone corresponds to a subcortical functional network during anticipation of pain – 3D Movie**

A significant subcortical network comprising 5 nodes and 6 edges in the high resting CVT group was apparent during anticipation of pain (FWER-corrected *p*<0.049). These nodes (coloured spheres) and edges (grey lines) are illustrated here in a 3-dimensional rotational movie. Nodes are color-coded as per the colour key. Abbreviations: Amyg, amygdala; Ant, anterior; L, left; Pall, pallidum; Sup, superior; Thal, thalamus.
